# Perceived stress and severity of depression mediate the association between alexithymia and suicidal ideation in patients with major depressive disorder

**DOI:** 10.1016/j.heliyon.2023.e21986

**Published:** 2023-11-03

**Authors:** Dan Li, Ying Gao, Shuhua Li, Chi Zhou, Yuting Wang, Meijuan Li, Nanage Guobule, Huan Mao, Xiangyang Zhang, Jie Li

**Affiliations:** a Laboratory of Biological Psychiatry, Institute of Mental Health, Tianjin Anding Hospital, Mental Health Center of Tianjin Medical University, Tianjin, 300222, China; b Department of Psychology, University of Chinese Academy of Sciences, Beijing, 100101, China; c Department of Clinical Psychology, Tianjin TEDA Hospital, Tianjin, 300457, China

**Keywords:** Alexithymia, Perceived stress, Suicidal ideation, Major depressive disorder

## Abstract

**Introduction:**

Alexithymia and perceived stress have been recognized as risk factors for suicide in patients with major depressive disorder (MDD). However, few studies have been conducted to examine the relationship between these factors.

**Methods:**

A cross-sectional study was conducted on 105 MDD patients. Alexithymia was assessed by the 20-Item Toronto Alexithymia Scale (TAS), perceived stress was assessed by the Perceived Stress Scale (PSS), severity of depression was assessed by the 17-item Hamilton Depression Rating Scale (HAMD), and suicidal ideation was assessed by the self-report Beck Scale for Suicide Ideation (SSI). A sequential mediation model was established to test the mediating effects of perceived stress and severity of depression on the association between alexithymia and suicidal ideation.

**Results:**

81 of the 105 participants (77.14 %) had suicidal ideation. Patients with suicidal ideation had greater difficulty in identifying feelings (DIF) (p = 0.046), higher severity of depression (p = 0.005) and perceived stress (p = 0.003). DIF subscale score of TAS was associated with perceived stress (r = 0.292, p = 0.003), severity of depression (r = 0.349, p < 0.001) and suicidal ideation (r = 0.229, p = 0.012). Sequential mediation model showed that perceived stress and severity of depression mediated the effect of DIF on suicidal ideation.

**Conclusions:**

This study suggests that perceived stress and severity of depression fully mediate the relationship between difficulty in identifying feelings and suicidal ideation in MDD patients.

## 1 Introduction

Suicide is a dangerous act in which individuals intentionally takes the means to end their lives under the influence of complex mental activity and is a major cause of death, with approximately 700,000 people dying by suicide each year [1]. Ninety percent of those who commit suicide suffer from a psychiatric disorder when they died [2,3]. Psychoanatomical studies suggest that nearly two-thirds of these psychiatric disorders are major depressive disorder (MDD) [4]. Approximately 1 in 6 patients with MDD eventually die by suicide despite treatment with antidepressants [5]. Given that suicidal ideation is the first step toward suicide [6,7], examining risk factors and interventions for suicidal ideation in MDD patients measures are essential for screening and prevention of suicidal behavior.

Alexithymia refers to a cognitive-emotional disorder in which people have difficulty identifying, describing, and expressing their feelings [8]. In addition, people with alexithymia feel a lack of imagination and fantasy, but rather externally oriented thinking [8]. Many studies have shown that alexithymia is associated with suicidal ideation in people with major depressive disorder [9,10] and is a significant predictor of subsequent suicidal ideation in the general population [11,12].

Several studies have shown that people with alexithymia have difficulty verbalizing their stress and coping with it appropriately [13,14], which may increase their feelings of isolation and loneliness. Research on responses to pressure conditions suggests that pressure is a human reaction to threatening and high-risk situations [15]. It is important to note that there is an important association that exists between perceived stress and general well-being [16]. According to the stress-alexithymia hypothesis [17], a lack of emotional awareness can hinder the recognition that an event is stressful. Stress is also thought to contribute to the onset of depression and to increase suicidal ideation in people with MDD [18].

One theoretical perspective that may explain the link between perceived stress, depressive symptoms, and suicidal ideation is Beck's cognitive theory of depression, in which Beck hypothesized a direct relationship between negative thinking and depressive symptoms. Consistent with this, life stressors and negative thinking about stressors may play a role in the development and maintenance of depressive symptoms, leading to an increased risk of suicide. Stress as a multidimensional psychological phenomenon has a variety of different aspects that need to be evaluated. Many studies on stress have focused only on the stressful event itself, without taking into account the degree to which individuals perceive stress, whereas the use of perceived stress scales can measure individuals' unpredictable, uncontrollable, or overwhelming feelings in the face of stressful events. In addition to being associated with depressive symptoms and suicidal ideation, perceived stress has also been found to be associated with alexithymia, and according to the stress-descriptive disorder hypothesis, a lack of emotional awareness hinders the recognition of stressful events [19].

Based on the results mentioned above, there may be a relationship between alexithymia, perceived stress, depression severity and suicidal ideation; however, the relationship between these factors has been less studied. The purpose of the present study was to assess the direct and indirect effects of alexithymia, perceived stress, and depression severity on suicidal ideation. We hypothesized that alexithymia affects suicidal ideation through the mediating effects of perceived stress and depression severity.

## 2 Methods

### 2.1 Study design and participants

This was a cross-sectional study in which 105 patients with MDD (male/female = 34/71) were recruited in Tianjin Anding Hospital started from May 2020 to March 2023, and patients were independently diagnosed by two experienced psychiatrists. The database included age, gender, nationality, education, and scores for alexithymia, perceived stress, severity of depression, and suicidal ideation.

Inclusion criteria were (1) age between 18 and 50 years; (2) meeting the diagnostic criteria for major depressive disorder according to the Diagnostic and Statistical Manual of Mental Disorders, Fifth Edition (DSM-5) [20]; and (3) first episode with no prior treatment. Exclusion criteria were (1) history of psychiatric disorders; (2) severe physical illness or brain injury, such as epilepsy, liver or kidney disease, or heart disease; (3) pregnancy or breastfeeding; and (4) suspected or confirmed history of alcohol or drug abuse.

The protocol received institutional approval from the Tianjin Anding Hospital Ethical Committee (ethics approval number: 2019-18) and was carried out in compliance with the Declaration of Helsinki. Signed informed consent was obtained before the start of the study.

### 2.2 Measurements

#### 2.2.1 Alexithymia

The severity of alexithymia was assessed using the 20-Item Toronto Alexithymia Scale [13], a widely used self-rating scale for alexithymia with 20 items. The total score is ranging from 20 to 100 points, with higher scores reflecting higher degrees of alexithymia. The TAS-20 has a three-factor structure: Factor one assesses the ability to identify and distinguish feelings (Difficulty in Identifying Feelings [DIF]); Factor two captured the ability to describe and express feelings to others (difficulty describing feelings [DDF]); while factor three evaluated the externally-oriented thinking (EOT).

#### 2.2.2 Perceived stress

The level of perceived stress of the participants were assessed using the 10-item Perceived Stress Scale (PSS) [21]. Ranging from 0 to 40, higher scores represent higher levels of perceived stress.

#### 2.2.3 Severity of depression

The severity of depression was evaluated using the 17-item Hamilton Depression Rating Scale (HAMD-17) [22]. HAMD-17 is a 17-item examiner-rating scale to quantify the severity of depressive symptoms [23]. The examiners of the scale were experienced psychiatrists, who had undergone consistent training before this study got started.

#### 2.2.4 Suicidal ideation

Suicidal ideation was assessed by the self-report Beck Scale for Suicide Ideation (SSI) [24]. The scale consists of 19 items that assess suicidal ideation. The SSI is scored by asking participants to accept the first five items. If the participant did not receive any score on the first five items, then the total score was zero (no thoughts). If the participants had suicidal ideation, the entirety of the scale needs to be completed to assess the intensity of the suicidal ideation. Ranging from 0 to 42, higher score indicated severer suicidal ideation.

### 2.3 Statistical analysis

Participants' characteristics were described by means, standard deviations (SD), and percentages. Kolmogorov-Smirnov test was used to examine the type of distribution of the variables. Differences in age, TAS, DIF, DDF, EOT, PSS, and HAMD scores between patients with and without suicidal ideation were compared using t-tests, and differences in gender and nationality were analyzed using chi-square tests. Pearson's correlation test was used to investigate the relationship between TAS, DIF, DDF, EOT, PSS, HAMD, and SSI scores. Bonferroni corrections were used to adjust for multiple testing. A test based on a two-tailed design with a p value < 0.05 was considered statistically significant.

After controlling for the effects of age, gender, and nationality, hypothetical sequential mediation models were developed to verify the mediating effect of perceived stress and depression severity from alexithymia to suicidal ideation. Regression coefficients were calculated in each mediation model. Bootstrapping was performed using 5000 resamples with 95 % confidence intervals to analyze the significance of the mediation models. Effects with CI excluding zero were interpreted as statistically significant.

All statistical analyses were performed with SPSS version 23.0 and PROCESS Macro 2.16 (IBM, Chicago, IL, USA).

## 3 Results

### 3.1 Characteristics of the patients

Patient characteristics were shown in Table 1. 81 of the 105 participants (77.14 %) had suicidal ideation. Patients with suicidal ideation were younger (p = 0.014), more males (p < 0.001), and had higher severity of depression and perceived stress (p < 0.001) compared to those without suicidal ideation. In addition, patients at risk of suicide had greater difficulty in identifying feelings (p = 0.044). There were no significant differences between the two groups in terms of TAS, DDF, EOT, education and nationality. However, the significant differences in age and difficulty in identifying feelings did not pass the Bonferroni corrections (Bonferroni corrected p < 0.05/7 = 0.0071).

**Table 1:** Demographic and characteristics of participants (N = 105).

Participant Characteristics	Total (N = 105)	Patients without suicidal ideation (N = 24)	Patients with suicidal ideation (N = 81)	P
Age (mean ± SD)	28.48 ± 9.981	32.17 ± 9.981	27.38 ± 7.663	0.014
Sex, male (n, %)	34 (32.4)	5 (20.8)	29 (35.8)	<0.001
Nationality, Han (n, %)	99 (94.3)	23 (95.8)	76 (93.8)	0.710
Education, years (mean ± SD)	14.45 ± 2.735	14.42 ± 3.476	14.46 ± 2.500	0.958
TAS (mean ± SD)	58.74 ± 10.387	55.13 ± 10.502	59.81 ± 10.171	0.052
DIF (mean ± SD)	22.03 ± 5.760	19.96 ± 5.996	22.64 ± 5.580	0.044
DDF (mean ± SD)	16.21 ± 3.529	15.58 ± 3.476	16.40 ± 3.545	0.325
EOT (mean ± SD)	20.50 ± 3.841	19.58 ± 3.161	20.78 ± 3.997	0.182
PSS (mean ± SD)	25.27 ± 5.154	21.92 ± 5.021	26.26 ± 4.787	<0.001
HAMD (mean ± SD)	18.61 ± 11.577	18.58 ± 5.594	23.31 ± 5.511	<0.001

TAS: Toronto Alexithymia Scale, DIF: Difficulty in Identifying Feelings, DDF: Difficulty in Describing Feelings, EOT: Externally-oriented thinking, PSS： Perceived stress scale, HAMD: Hamilton Depression Rating Scale, SSI: Beck scale for suicide ideation.

### 3.2 Associations between alexithymia, perceived stress, severity of depression and suicidal ideation

The results of the correlation analysis were shown in Table 2. Alexithymia was positively associated with perceived stress (β = 0.217, p = 0.026), depression severity (β = 0.248, p = 0.011) and suicidal ideation (β = 0.227, p = 0.020). As for the three subfactors of alexithymia, only the difficulty in identifying feelings (DIF) score was associated with perceived stress (β = 0.314, p = 0.001), depression severity (β = 0.356, p < 0.001) and suicidal ideation (β = 0.281, p = 0.004). Perceived stress was positively associated with depression severity (β = 0.351, p < 0.001) and suicidal ideation (β = 0.323, p = 0.001). Severity of depression was also positively associated with suicidal ideation (β = 0.435, p < 0.001). The correlation analysis of DIF, perceived stress, depression severity and suicidal ideation passed the Bonferroni corrections (Bonferroni corrected p < 0.05/6 = 0.0083).

**Table 2:** Pearson correlation coefficients between alexithymia (TAS, DIF, DDF, EOT), perceived stress (PSS), severity of depression (HAMD) and suicidal ideation (SSI).

	TAS	DIF	DDF	EOT	PSS	HAMD	SSI
TAS	1						
DIF	0.879***	1					
DDF	0.822***	0.656***	1				
EOT	0.631***	0.274**	0.321**	1			
PSS	0.217*	0.314**	0.163	−0.034	1		
HAMD	0.248*	0.356***	0.97	0.48	0.351***	1	
SSI	0.227*	0.281**	0.148	0.055	0.323**	0.435***	1

*p < 0.05, **p < 0.01, ***P < 0.001. TAS: Toronto Alexithymia Scale, DIF: Difficulty in Identifying Feelings, DDF: Difficulty in Describing Feelings, EOT: Externally-oriented thinking, PSS: Perceived stress scale, HAMD: Hamilton Depression Rating Scale, SSI: Beck scale for suicide ideation.

### 3.3 Sequential mediating effects between DIF, PSS, HAMD, and SSI

**Fig. 1:**
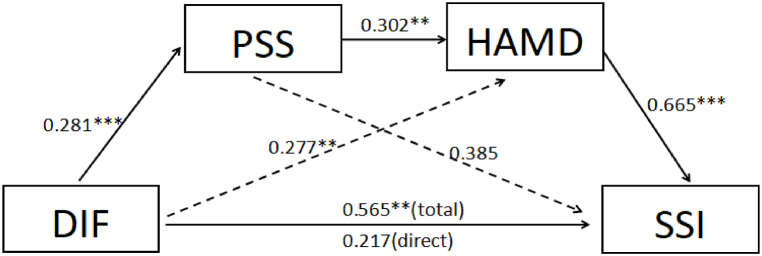
Examined sequential mediation model perceived stress and severity of depression on alexithymia and suicidal ideation. *p < 0.05, **p < 0.01, ***P < 0.001. Solid lines represent significant pathways, the dashed line represents a non-significant pathway. Unstandardized coefficients are presented. DIF: Difficulty in Identifying Feelings; PSS: Perceived stress scale; HAMD: Hamilton Depression Rating Scale; SSI: Beck scale for suicide ideation.

The results of the regression analysis of the mediation model were shown in Fig. 1. Because DDF and EOT scores were not related to PSS, HAMD, and SSI scores, mediation models involving these two factors could not be developed. Regarding DIF, the paths from DIF to PSS (β = 0.281, p < 0.001) and HAMD (β = 0.277, p = 0.004) were positive and significant. There was no significant correlation between PSS and SSI (β = 0.385, p = 0.070) after HAMD was controlled as mediator. There was a significant correlation between PSS and HAMD (β = 0.302, p = 0.004). Also, HAMD was significantly correlated with SSI (β = 0.665, p < 0.001).

Table 3 showed the bootstrap analysis of sequential mediation effects. There was a significant mediation effect of PSS on the relationship between DIF and suicidal ideation (95 % CI 0.0038–0.2271); a significant mediation effect of HAMD on the relationship between DIF and suicidal ideation (95 % CI 0.0673–0.3687). The DIF had an indirect effect on the suicidal ideation with the sequential mediating effect of PSS and HAMD (95 % CI 0.0140–0.1548). The total effect of DIF on suicidal ideation was significant (β = 0.565, p = 0.007, 95 % CI 0.1550–0.9753), but when perceived stress and depression severity were controlled for as mediators, the direct effect was not significant (β = 0.217, p = 0.317, 95 % CI -0.2107–0.6438). Thus, perceived stress and depression severity acted as full mediators in this sequential mediation model.

**Table 3:** The sequential mediating effects among variables.

Mediating Pathway	Bootstrapping Estimate		95 % CI	
	Effect	SE	LLCI	ULCI
DIF ⇒ Perceived stress⇒ suicidal ideation	0.1081	0.0678	0.0038	0.2771
DIF ⇒ severity of depression⇒ suicidal ideation	0.1841	0.0746	0.0673	0.3687
DIF ⇒ Perceived stress⇒ severity of depression⇒ suicidal ideation	0.0564	0.0335	0.0140	0.1548

CI: confidence interval; LLCI: lower limit confidence interval; ULCI: upper limit confidence interval; SE = standard error; DIF: difficulty in identifying feelings.

## 4 Discussion

In this clinical sample of 105 patients with MDD, we found that alexithymia, perceived stress and severity of depression were positively associated with suicidal ideation. Furthermore, the effect of alexithymia on suicidal ideation was mediated by perceived stress and severity of depression.

### 4.1 Alexithymia and suicidal ideation

Many studies had shown an association between alexithymia and suicidal ideation in MDD and non-MDD populations [9,12,[25], [26], [27], [28], [29]]. A longitudinal study had shown that alexithymia is a substantial predicting factor for subsequent suicidal ideation in general population [11,12]. Furthermore, among 145 outpatients with MDD, alexithymia was considered an independent predictor of suicidal ideation [10].

The number of people reporting suicidal ideation might be lower than the actual number due to the stigma associated with depression and suicide, which might add extra risk to that already vulnerable group of patients. Our findings suggested that alexithymia might be a potential predictor of suicidal ideation and was less threatening when asked. Furthermore, the results showed greater correlations between perceived stress, depression severity, suicidal ideation and the difficulty in identifying feelings (DIF) subscale compared to the total TAS score, while the subscales of difficulty in describing feelings (DDF) and externally oriented thinking (EOT) exhibited no significance, which was also in line with previous studies [10]. Therefore, DIF might constitute the main component of alexithymia that influenced suicidal ideation.

### 4.2 DIF, perceived stress, and suicidal ideation

In addition to being associated with suicidal ideation, alexithymia was also associated with perceived stress, which is consistent with previous research [15]. And perceived stress in MDD could enhance suicidal ideation [18]. In our study, the mediation model from DIF to suicidal ideation showed significance through perceived stress, suggesting that DIF affects suicidal ideation by increasing perceived stress in MDD patients. Thus, patients' difficulties in identifying their feelings might suppress their emotional regulation and inappropriately cope with stress, thus exacerbating their suicidal ideation.

### 4.3 DIF, depression severity, and suicidal ideation

Although earlier studies have consistently demonstrated that depression is highly related to suicide [30,31], risk factors for suicide in people who have been diagnosed with depression are complicated and the severity of depression on its own is not accurate enough to predict it [32]. In our study, the mediation model from DIF to suicidal ideation showed significance through depression severity, suggesting that DIF influenced depression severity thereby exacerbating suicidal ideation in MDD patients. However, there was an ongoing debate as to whether alexithymia was a state or trait-dependent phenomenon [33,34]. Several studies had suggested that alexithymia is a stable feature [[35], [36], [37]], while a few conflicting findings suggested that alexithymia was a state-dependent phenomenon and fluctuated with simultaneous changes in depression severity [34,38]. Therefore, the direct causal relationship between alexithymia and depression remains unclear and future research is needed.

### 4.4 Sequential mediation model

The results of the sequential mediation model showed that the direct effect of DIF on suicidal ideation was no longer significant after controlling for perceived stress and severity of depression as mediating factors. In contrast, the sequential mediation model (DIF ⇒ perceived stress ⇒ severity of depression ⇒ suicidal ideation) was significant. Thus, perceived stress and severity of depression acted as full mediators in this sequential mediation model. Supporting our hypothesis, our results suggested that DIF, perceived stress, and severity of depression might constitute a causal relationship rather than cause suicidal ideation separately. For patients with MDD, DIF did not directly cause suicidal ideation, but rather through perceived stress and severity of depression. The difficulty in identifying feelings made patients more stressed in the face of the event, and the more stressed, the more the severity of depression appeared, and the depression would lead to suicidal ideation.

The results may provide an identification tool for assessing suicidal ideation in the MDD population, which may then reduce suicidal behavior or be a treatment target for depression or suicide. Furthermore, of the three aspects of alexithymia, it was the difficulty in identifying feelings that affected perceived stress, depression severity, and suicidal ideation; therefore, psychotherapy and antidepressants to improve identification of feelings are necessary. Furthermore, the association between difficulty in identifying feelings and suicidal ideation was mediated by perceived stress and depression severity in that order. To reduce suicidal ideation in patients with MDD, comprehensive treatment and prevention strategies need to be considered. When considering therapeutic interventions, one study suggested that serotonergic antidepressants might be associated with difficulty recognizing feelings, and difficulty recognizing feelings could be considered a side effect of antidepressants [39]. Therefore, in addition to being a predictor of suicidal ideation, alexithymia may also be used as a reference for the selection of antidepressants. Difficulties in identifying antidepressant-induced feelings can in turn exacerbate the severity of depression and thus affect the efficacy of antidepressants. In addition, in terms of drug selection, suicidal behavior may be associated with associated neurobiological dysfunction, particularly immune-inflammatory abnormalities [40], new strategies are needed to successfully address those who do not respond or partially respond to existing pharmacotherapy and are at high risk for negative clinical outcomes. Importantly, there is evidence that at low doses, some psychoactive compounds are effective, well-tolerated, and safe options in reducing depressive symptoms and severe suicidal ideation, even in patients with treatment-resistant depression and a higher risk of suicide associated with immune inflammatory dysfunction [41].

This study has its limitations. The sample size was small, so the findings should be interpreted and generalized to a larger population in the future. Also, the third item of the HAMD scale is the suicide risk assessment, which may affect the results of the correlation between depression severity and suicidal ideation. However, we found that some patients without suicidal ideation had high HAMD scores, while some patients with suicidal ideation had low HAMD scores, so we did not remove the third item when calculating the HAMD score. In addition, the current study assessed only suicidal ideation, limiting the ability to examine the role of alexithymia in suicide attempts or self-harm. Furthermore, this is a cross-sectional study and it remains unclear how these factors (alexithymia, perceived stress, severity of depression, and suicidal ideation) change over time in response to the intervention and whether these factors are stable or state-based. Thus far, we cannot exclude that depressive episodes may be precursors to bipolar or other psychiatric disorders. Furthermore, the severity of depression was analyzed by clinician rating scales, whereas alexithymia and suicidal ideation were assessed by self-rating scales, and bias may arise from the nature of the self-rating scales. Taken together, these limitations may restrict the generalization of our results. In addition, the effect sizes, while statistically significant, are at a low level. The reason may be that there are other variables which also play a role but not been considered, such as childhood trauma [42]. As such there are many other factors that deserve to be further explored in the future. It is also possible that the mediator's effect is subtle and only works in specific populations, rather than in the entire population of patients with major depressive disorder. Nevertheless, even small effect sizes may have real impacts, especially in particular populations. While larger effect sizes may be more meaningful, smaller effect sizes can also provide some insights into the subtleties and nuances of the various factors that play a role in depression. Finally, other factors, such as harm avoidance and bereavement, had also been associated with impaired psychological functioning and elevated risk of suicide. Further research should take these factors into account.

Given these limitations, this is the first study to examine the role of perceived stress and depression severity on the association between alexithymia and suicidal ideation in MDD patients to our knowledge. This study added empirical data to the literature on factors associated with suicidal ideation in MDD patients. The results of the present study offer some possibilities for predicting suicidal ideation. We found that difficulties in identifying feelings could cause people to perceive more stress, which lead to emotional problems. The resulting emotional problems will lead to more serious consequences, and correctly identifying feelings might play a more important role in mental health than we might think. In clinical work, helping people with MDD learn to identify their emotions may be an effective way to relieve stress, improve depressive symptoms, and sequentially alleviate suicidal ideation. Today, depression and suicide are thrust into the limelight, placing a tremendous psychosocial and economic burden on society and healthcare systems. Knowing the specific contributors related to suicidal ideation may help in prevention and treatment. In addition, improving the ability to identify feelings and change perceptions of stress are expected to improve quality of life and functional outcomes for people with MDD. However, our study lacked follow-up data. Long-term follow-up for explicit assessments of mood state and alexithymia, as well as follow-up after clinical remission and pharmacotherapy, are needed to generalize the current results.

## 5 Conclusions

The sequential mediation model suggested that perceived stress and depression severity fully mediated the relationship between difficulties in identifying feelings and suicidal ideation in patients with MDD. Therefore, psychiatrists should recognize patients' difficulties in identifying feelings in order to establish strategies to prevent depression and suicidality. Future research needs to develop appropriate interventions that address these mediators and find effective mechanisms for integration with clinical practice.

## Funding

This study was financially supported by the Tianshui Chengji Star Talent Project and Tianjin Scientific and Technological Talent Cultivation Project (No. KJ20025). These sources had no further role in this study design, data collection, and statistical analysis, drafting of the report, and submitting the paper for publication.

## Data availability statement

Data associated with the study has not been deposited into a publicly available repository. Data are available on request through the authors’ direct contacts, under some terms and conditions.

## CRediT authorship contribution statement

**Dan Li:** Writing – original draft, Methodology. **Ying Gao:** Funding acquisition. **Shuhua Li:** Data curation. **Chi Zhou:** Data curation. **Yuting Wang:** Data curation, Conceptualization. **Meijuan Li:** Writing – review & editing. **Nanage Guobule:** Data curation. **Huan Mao:** Data curation. **Xiangyang Zhang:** Writing – review & editing. **Jie Li:** Writing – review & editing, Supervision.

## Declaration of competing interest

The authors declare that they have no known competing financial interests or personal relationships that could have appeared to influence the work reported in this paper.
